# Phase I Trial of Pyragrel, a Novel Thromboxane Synthetase Inhibitor, to Evaluate the Safety, Tolerability, and Pharmacokinetics in Healthy Volunteers

**DOI:** 10.3389/fphar.2019.01231

**Published:** 2019-10-24

**Authors:** Chan Zou, Xiaocong Zuo, Jie Huang, Ye Hua, Shuang Yang, Xiaoyan Yang, Can Guo, Hongyi Tan, Jun Chen, Zhaoxing Chu, Qi Pei, Guoping Yang

**Affiliations:** ^1^Center of Clinical Pharmacology, The Third Xiangya Hospital, Central South University, Changsha, China; ^2^Department of Pharmacy, The Third Xiangya Hospital, Central South University, Changsha, China; ^3^Department of Pharmacy, The First Affiliated Hospital of Xinjiang Medical University, Urumqi, China; ^4^Innovative Drug Design and Evaluation Center, Hefei Industrial Pharmaceutical Institute Co., Ltd, Anhui, China

**Keywords:** phase I trial, pyragrel, pharmacokinetics, pharmacodynamics, safety

## Abstract

**Background and Objective:** Inhibition of thrombosis and platelet aggregation through a thromboxane synthetase inhibitor proved to be an effective and promising treatment for cardiovascular and/or cerebrovascular disease (CCVD) patients. This phase I study evaluated the safety, tolerability, and pharmacokinetics of sodium pyragrel, a novel thromboxane A_2_ synthetase inhibitor, in healthy volunteers.

**Methods:** A total of 84 healthy Chinese volunteers were enrolled in the study and randomized into one of five dosing regimens of intravenous pyragrel, which were single ascending dose (30 to 300 mg), multiple doses (pyragrel 180 mg once daily on Day 1 and Day 6, twice daily from Day 2 to Day 5), 3×3 Latin square crossover (60, 120, 240 mg), and a continuous dose (360 mg in 24 h), respectively. Plasma concentrations were determined using HPLC-MS/MS. Pharmacokinetics parameters were calculated with non-compartment analysis.

**Results:** The maximum plasma concentrations of pyragrel were essentially reached at the end of the 3 h infusion. The pharmacokinetic process of pyragrel and two main metabolites (BBS and BJS) is linear over the 30–300 mg dose range, with no significant accumulation on multiple doses. The urinary excretion of pyragrel accounted for more than 70% of the total drug amount. Preliminary pharmacodynamic results demonstrated that the production of urinary 11-D-HTXB_2_ was time- and dose-dependently inhibited by single i.v. dose of pyragrel.

**Conclusions:** Pyragrel was well tolerated after single ascending doses up to 300 mg, multiple doses of 180 mg, and continuous administration of 360 mg within 24 h. No drug-related, serious adverse drug reactions occurred during the five-part study. The most common pyragrel-related adverse events (AEs) were total bilirubin (TB)/direct bilirubin (DB) elevations with a relatively low incidence rate and seemed to be dose independent. Given the acceptable safety and appropriate pharmacokinetic properties of sodium pyragrel proven in this study, continued clinical development is warranted. The study was registered at http://www.chictr.org.cn (ChiCTR-IID-16010159).

## Introduction

Thromboembolism, which is commonly induced by platelet aggregation and vasocontraction, causes vascular damage and leads to extremely poor outcomes in cardiovascular and/or cerebrovascular disease (CCVD) patients (Organization World Health, 2007). In China, more than 2.6 million people die from CCVD each year, and 75% of survivors are disabled after chemotherapeutic or surgical treatment, among which more than 40% are severely disabled (Li and Ge, 2015). Except for surgical intervention being used in certain circumstances (e.g., severe conditions or with appropriate indications for surgery), the most recommended therapy for CCVD patients is most likely based on chemotherapeutic agents, which have been demonstrated to be highly effective (Fleg et al., 2011). These chemotherapeutics include antiplatelet drugs (Michelson, 2010), antithrombotic agents (Donnan et al., 2011), vasodilators (Wei et al., 2018), and other cardiovascular protectants (Satoh et al., 2007). Sodium ozagrel developed as a thromboxane A2 synthetase inhibitor that functions through inhibition of thrombosis and platelet aggregation and is considered as the only one treatment for patients with acute thrombotic cerebral infarction complicated with or without dyskinesia; however, the incidence rate of severe bleeding is up to 5% in patients with ozagrel treatment (Morio et al., 1993).Therefore, urgent requirements for developing innovative drugs are especially encouraged and challenged in China, since China has a very large population of cardiovascular patients whose treatment is in sharp conflict with the extreme lack of intellectual property protection and innovative cardiovascular drugs (Wu et al., 2016).

Recently, a new thromboxane synthetase inhibitor, sodium pyragrel, was developed as an analog of ozagrel, which exerts significant antiplatelet aggregation and antithrombotic effects through TXA_2_ inhibition (Bao et al., 2012; Fang et al., 2014). This new drug was synthesized with two well-known antithrombotic small molecule compounds (Xie et al., 2015), the Chinese herbal medicine ligustrazine and ferulic acid (Srinivasan et al., 2007; Yu et al., 2016). Due to the significant inhibitory activity against platelet aggregation and thrombosis, pyragrel exhibited markedly protective effects against focal cerebral ischemic injury and focal cerebral ischemia-reperfusion in well-established rat models of focal cerebral ischemia (Wang and Wu, 2014). In addition, significant improvements in nerve function were observed *in vivo* after continuous infusion of pyragrel for 7 days in rats with focal cerebral ischemia-reperfusion injury, and significant protective effects against *in vitro* inflammatory induced human umbilical vein endothelial cells (HUVEC) injury also exerted.

Additionally, long-term toxicity studies further demonstrated that pyragrel has superior efficacy and safety profiles compared to ozagrel. After administration intravenously of sodium pyragrel up to 40 mg/kg/day for 3 months to beagle dogs, safety conclusions revealed that the maximum safe dose of pyragrel is much higher than that of sodium ozagrel, 12.5 mg/kg/day. Moreover, the prolongation of coagulation time was comparable between pyragrel and ozagrel in mice receiving daily 36 mg/kg injections for 4 days, indicating similar delayed bleeding risk in the two treatment groups, but the bleeding time was 35% lower in pyragrel treated mice (P < 0.01).

Here, we aimed to evaluate the safety, tolerability, and pharmacokinetics of intravenous pyragrel in healthy volunteers.

## Materials And Methods

This phase I study used a randomized, double-blind, active drug (sodium ozagrel) and placebo control (saline) (Part I, single ascending dose) or open-label (Parts II-V) design to evaluate the safety, tolerability, pharmacokinetics, and preliminary pharmacodynamics of sodium pyragrel in healthy volunteers. The study was conducted at a single center in China (The Third Xiangya Hospital, Changsha, China).

### Study Design

**Figure 1:**
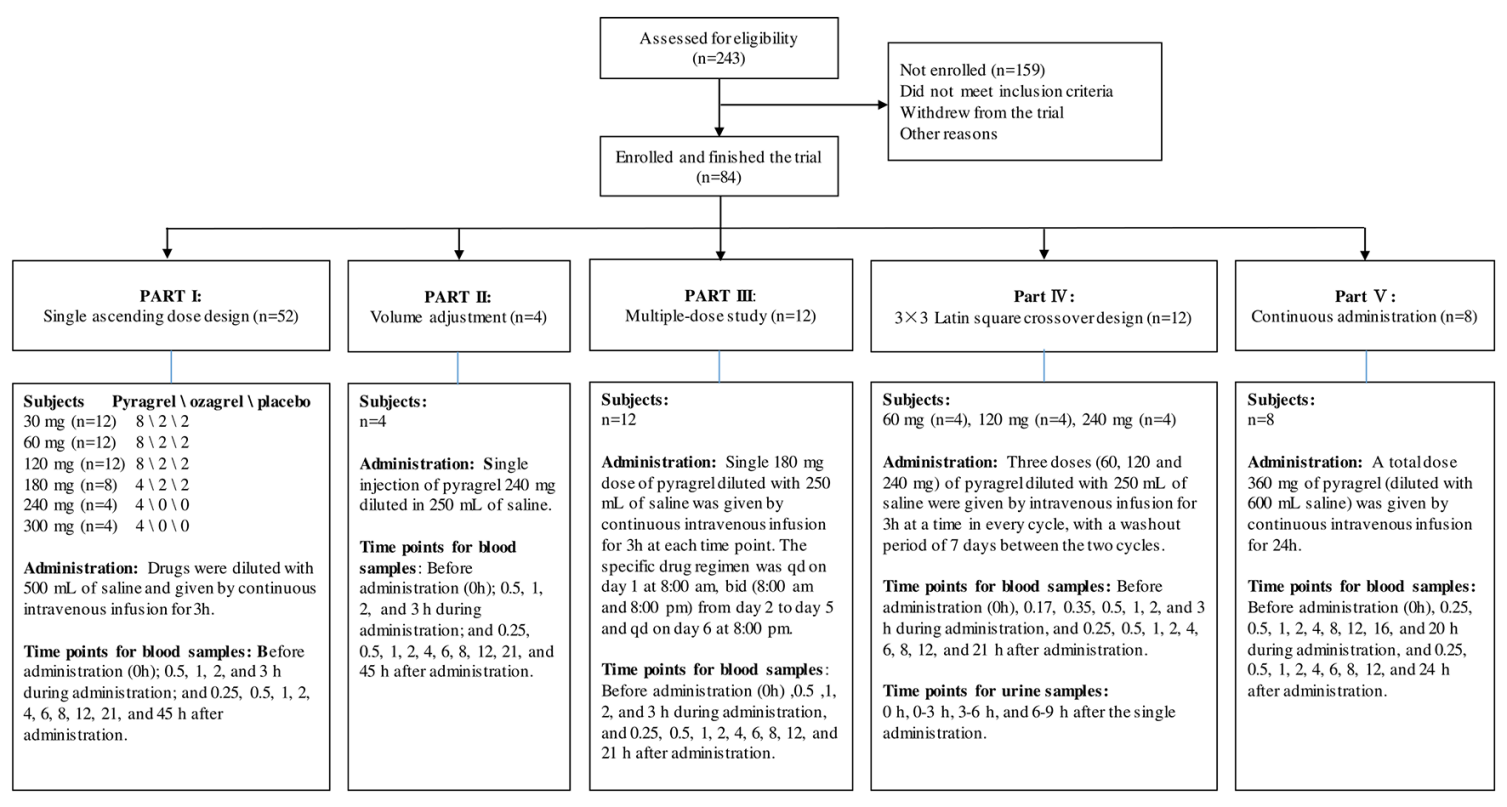
Flow chart of the pyragrel phase I trial study. Flow chart illustrated the study design, clinical screening procedure, information about the number of subjects enrolled, drug administration methods, predetermined time points for blood and urine samples collection in Part I–V.

For this five-part (Parts I–V) first-in-human phase I study conducted in healthy adult volunteers, the detailed inclusion and exclusion criteria for the screening process were consistent in all parts of the study and are listed in **Table S1**. During the screening period (1 week before administration), subjects received an exhaustive health examination, and the recorded items and tests included demographic characteristics, physical examination, vital signs, electrocardiogram, chest X-ray examination, blood type, routine blood, routine urine, routine stool, fecal occult blood, liver and kidney function, blood glucose, blood lipid, plasma electrolytes, myocardial enzymes, coagulation function, four pre-transfusion tests [hepatitis B virus surface antigen (HBsAg), antibody against hepatitis C virus (anti-HCV), antibody against human immunodeficiency virus type 1/2 (anti-HIV), antibody against Treponema pallidum (anti-TP)] and pregnancy examination (applied to female volunteers only). Eligible subjects were randomly assigned to receive intravenously administered pyragrel, ozagrel, or placebo. The flow chart of the five-part phase I study is shown in **Figure 1**.

### Safety Assessments

Safety was assessed using subject interviews and adverse event (AE) monitoring. All subjects received laboratory examinations after termination of drug infusion for 48 or 72 h; the examination items are the same as those listed in the screening process. If clinical abnormalities were present, further follow-ups were required until the laboratory test values or vital sign levels of the abnormal items returned to normal values or stable levels. The clinical significance of abnormal laboratory test values was determined by the physicians.

### Dose Regimens and Pharmacokinetic Analysis of Blood and Urine Samples

Pyragrel administration regimens are as follows: In the single-escalating-dose study (Part I), a total of 52 subjects are enrolled to receive one of three treatments (pyragrel, ozagrel, or placebo, diluted in 500 Ml normal saline) as a 3-h i.v. infusion. Six dose levels of pyragrel (30, 60,120, 180, 240, 300 mg) were intravenously infused to healthy subjects. Of the 12 subjects each at the first three dose groups (30, 60, and 120 mg), 8 received pyragrel, 2 received ozagrel (80 mg), and 2 received placebo. Eight subjects were assigned as 4/2/2 for pyragrel, ozagrel, and placebo at the 180 mg level. Only four subjects were enrolled at the 240- and 300-mg group each; all received pyragrel. Escalation to the next dose level was applied after safety and tolerability were demonstrated. In the saline-volume-changed study (Part II), the safety and pharmacokinetics of pyragrel were assessed by decreasing the volume of dilution saline. Four subjects received a single 3 h infusion of pyragrel 240 mg diluted in 250 ml saline instead of the 500 ml used in Part I. Blood samples were collected at multiple time points before and after the initiation of the infusion in Part I and Part II; 15 blood samples were predetermined as follows: before drug administration (time 0), 0.5, 1, 2, 3 (4 samples collected during infusion), 3.25, 3.5, 4, 5, 7, 9, 11, 15, 24, and 48 h (10 samples collected post infusion) after the initiation of the infusion. In the multiple-dose study (Part III), 12 subjects received a single 3 h infusion of 180 mg pyragrel (diluted in 250 ml saline) on Day 1 and Day 6 and twice 3 h infusion of 180 mg pyragrel from Day 2 to 5; 14 blood samples were collected on Day 1 and Day 6 before drug administration (0 h), 0.5, 1, 2, 3, 3.25, 3.5, 4, 5, 7, 9, 11, 15, 24 h after the initiation of the infusion; moreover, 5 ml cubital vein blood was collected each time right before pyragrel infusion on Day 2 to Day 5. In the 3×3 crossover study (Part IV), 12 subjects were randomized to receive each of the three dosing groups (60, 120, or 240 mg) of pyragrel (diluted with 250 ml saline, 3 h infusion) per period, with a 7-day washout period between doses. Sixteen blood samples were collected as follows: before drug administration (0 h), 0.17, 0.35, 0.5, 1, 2, 3, 3.25, 3.5, 4, 5, 7, 9, 11, 15, and 24 h after the initiation of the infusion at each period. In the prolonged infusion study (Part V), four subjects received continuous 24 h infusion of pyragrel 360 mg (diluted in 600 ml saline); 19 blood samples were collected as follows: before drug administration (0 h), 0.25, 0.5, 1, 2, 4, 8, 12, 16, 20 (nine samples collected during administration), 24.25, 24.5, 25, 26, 28, 30, 32, 36, and 48 h after the initiation of the infusion (details shown in **Figure 1**). In all five cohorts, 5 ml of cubital vein blood was collected with a sodium heparin anticoagulant tube and centrifuged at 3000 r·min^-1^. Each plasma sample was divided into two aliquots and stored at −20°C until further bioanalysis. The HPLC-MS/MS method was used to determine the plasma concentrations of pyragrel, BBS (propionic acid metabolite of pyragrel), and BJS (benzoic acid metabolite of pyragrel) in the plasma as described previously (Yang et al., 2018). Briefly, 10 µl of the extract drug from human serum was injected onto a Zorbax EcLipse XDB C18 column (2.1 mm × 150 mm, 5 μm) (Agilent, USA) using isocratic elution with a mobile phase composed of methanol, water, and formic acid (65:35:0.1, v/v/v). Determination of the analytes was achieved by tandem-mass spectrometry with positive electrospray ionization. The ranges of quantification were 0.5–1,000 ng/ml for pyragrel and BBS, and 0.25–500 ng/ml for BJS in serum.

Urine samples were collected after the initiation of pyragrel infusion as follows in Part IV: 0–3 (3 h infusion period), 3–7, 7–11, 11–15, and 15–24 h after pyragrel administration during each period. The concentrations of pyragrel and four metabolites (BBS, BJS, MW318, MW328) were determined by solid-phase extraction coupled with high-performance liquid chromatography tandem mass spectrometry, followed by macroporous resin-based purification. The ranges of quantification were 0.1–100, 0.1–100 μg/ml, and 0.2–100 μg/ml for pyragrel, BBS, and BJS in urine, respectively (Zhao et al., 2017). Production of 11-dehydro-thrombxane B2 (11-D-HTXB2) in urine sample was detected by ELISA method.

### Data Analysis

Pharmacokinetic parameters were calculated using the noncompartmental model (NCA module), WinNonlin 6.1 (Pharsight Corporation, Mountain View, CA, USA). Statistical analysis was performed with SPSS 18. The actual dosing and sampling times were utilized in the analysis. Pharmacokinetic parameters were summarized by dose regimens using descriptive statistics, including n, mean, and the coefficient of variation (CV). For Tmax, only n, min, median, and max were reported.

The vital signs were analyzed by repeated measures analysis of variance (ANOVA), and laboratory indexes were analyzed by the paired t-test and one-way ANOVA. Comparison within different dose regimens of pyragrel or among different treatments (pyragrel, ozagrel, and placebo) or production of urinary 11-D-HTXB2 among groups was performed with the t-test, and expected values were considered along with the actual situation. Descriptive methods were used to compare the incidence of AE between pyragrel and ozagrel/placebo or in different pyragrel dose regimens; the normal/abnormal changes in laboratory examinations before and after administration were described.

In Part I, dose proportionality was evaluated using linear regression analysis of Cmax and AUC versus dose, the ln–ln plots of AUClast, AUCinf, and Cmax versus dose were generated, and the 95% confidence intervals (CIs) of the slopes that included the value of 1 indicate dose proportionality.

In Part III, the accumulation of pyragrel after administration of multiple doses of pyragrel 180 mg was determined by calculating accumulation factor R. R was defined as the AUC_TAU,multiple_/AUC_TAU,single_, where AUC_TAU,mutiple_ is the area under the concentration-time curve from time zero to the end of the dosing period at steady state. AUC_TAU, single_ is equal to AUC_INF_ for a single dose.

In Part IV, a multivariate ANOVA model containing subjects, treatments (60, 120, or 240 mg), periods, and gender was used to analyze log-transformed Cmax and AUC. Tmax was analyzed using various related Friedman nonparametric tests.

## Results

### Demographic Characteristics

A total of 84 healthy Chinese subjects (42 males and 42 females) were enrolled from May 27, 2014 to February 4, 2016. All subjects completed the study according to the protocol. **Tables 1** and **2** summarize the baseline demographics of all participants. There was no significant difference in age, height, weight, and body mass index (BMI) among all groups.

**Table 1:** Demographic variables of healthy volunteers in Part I.

Dose groups	Number (*n*)	Age (Years)	Height (m)	Weight (kg)	BMI (kg/m^2^)
30 mg	8	23 ± 4	1.69 ± 0.08	59.3 ± 7.6	20.6 ± 1.1
60 mg	8	22 ± 4	1.65 ± 0.05	57.2 ± 6.4	20.9 ± 1.7
120 mg	8	24 ± 4	1.67 ± 0.07	59.7 ± 7.7	21.5 ± 2.0
180 mg	4	21 ± 3	1.71 ± 0.06	60.3 ± 6.1	20.6 ± 1.4
240 mg	4	24 ± 6	1.62 ± 0.14	56.3 ± 7.0	21.5 ± 1.2
300 mg	4	23 ± 3	1.65 ± 0.09	60.0 ± 4.2	22.1 ± 1.2
Ozagrel	10	24 ± 5	1.65 ± 0.09	58.9 ± 9.2	21.6 ± 1.6
Placebo	10	22 ± 3	1.62 ± 0.08	53.5 ± 5.6	20.3 ± 1.2
Total	56	23 ± 4	1.65 ± 0.08	57.7 ± 7.1	21.1 ± 1.5
P		0.803	0.651	0.378	0.201

**Table 2:** Demographic variables of healthy volunteers in Parts II–V.

Variables	Part II	Part III	Part IV	Part V
Total Number	4	12	12	4
Sex (%)				
Male	2 (50%)	6 (50%)	6 (50%)	2 (50%)
Female	2 (50%)	6 (50%)	6 (50%)	2 (50%)
Age, Years	24 ± 4	22 ± 3	24 ± 2	23 ± 3
Height, m	1.66 ± 0.10	1.63 ± 0.11	1.62 ± 0.09	1.63 ± 0.09
Weight, kg	62.1 ± 8.0	56.4 ± 9.7	56.4 ± 7.4	557.1 ± 10.5
BMI, kg/m^2^	22.4 ± 0.7	21.0 ± 1.8	21.5 ± 1.8	22.8 ± 1.8

Data are the mean ± SD, except sex (male/female), which is in %; BMI, body mass index; SD, standard deviation.

### Safety Evaluation Of Pyragrel

Eighty-four participants were included in the safety evaluation; all of them were randomized or recruited to receive one of the two active treatments (sodium pyragrel, sodium ozagrel) or placebo. No severe or life-threatening AEs were observed, and no participant dropped out during the study period. The types and incidence rate (IR) of AEs are summarized in **Table 3**.

**Table 3:** Summary of AE patterns in each part of the study.

	Pyragrel	AE Type	Ozagrel/placebo	AE Type
Part I				
30 mg	2	DB elevation, palpitation	1	DB elevation
60 mg	2	Leucocytes increase, positive urine protein	0	0
120 mg	1	DB elevation	1	DB elevation
180 mg	3	DB elevation (2), ventosity	1	abdominal pain
240 mg	2	APTT extension	0	0
300 mg	2	Lower hemoglobin, total bile acid increase	0	0
Part II	2	DB/TB elevation	N/A	N/A
Part III	12	DB/TB elevation, alanine aminotransferase elevated, abdominal pain, headache, low blood potassium, urine frequency, fecal OBT+.	N/A	N/A
Part IV	8	Dizziness, TB/DB elevation, transient left tinnitus, double upper arm papules, fatigue	N/A	N/A
Part V	1	DB elevation	N/A	N/A
Total	35		3	

TB, total bilirubin; DB, direct bilirubin; OBT, fecal occult blood test, APTT, activated partial thromboplastin time. N/A, not applicable.

The overall AE incident rate was comparable between pyragrel and ozagrel; no placebo-related AEs were reported. Among 52 enrolled subjects in Part I, 12 AEs occurred in 36 pyragrel-treated subjects (12/36, IR 33.3%) and 3 AEs in 8 subjects of ozagrel treatment (3/8, IR 12.5%). There is no statistically significant difference of IR between treatments (P=0.882). Moreover, AEs among pyragrel-treated group did not appear to be dose-dependent and most AEs were mild in intensity. Reported AEs in Part I were activated partial thromboplastin time (APTT) extension, elevations in direct bilirubin (DB) and total bilirubin (TB), bellyache, ventosity, palpitations, positive for fecal occult blood test (OBT+), etc.

In the saline-volume changed study, the dilution volume of saline decreased from 500 ml (used in Part I) to the generally used 250 ml in the clinic. Pyragrel (240 mg) was dissolved in 250 ml saline intravenously infused for 3 h in four subjects. Two subjects showed elevated serum bilirubin (1 DB, 1 TB), suggesting that a decreased volume of diluted saline may probably increase the incidence of AE (2/4, IR 50%).

AE patterns reported following multiple doses of pyragrel in Part III were similar to those in Part I after single dose, and incidence as well (12/36, 33.3% versus 33.3%). Continuous infusion of pyragrel showed a lower incidence than single dose (1/4, 25.0% for Part V versus 33.3% for Part I pyragrel). All AEs were well tolerated and subjects recovered fully without treatment from all AEs.

### Pharmacokinetic Properties Of Pyragrel

**Figure 2:**
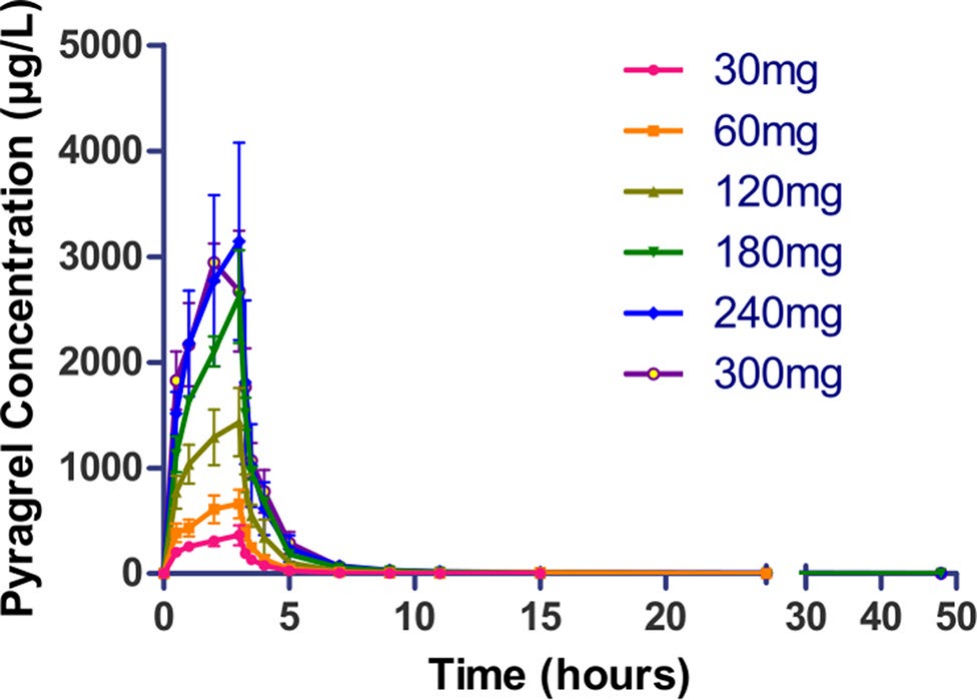
Mean (±SD) concentration-time profiles of pyragrel following a 3 h intravenous infusion of single ascending doses of pyragrel (30, 60, 120, 180, 240, 300 mg) in healthy volunteers (Part I). Fifty-two participants randomized to receive i.v. 3 h infusion of single dose of pyragrel ascendingly (30, 60, 120, 180, 240, 300 mg), ozagrel 80 mg, or placebo diluted in 500 ml saline; pyragrel concentrations of blood samples were determined over 48 h after the initiation of the infusion.

Following a 3 h intravenous infusion of single ascending doses of pyragrel (30, 60, 120, 180, 240, or 300 mg), the maximum plasma concentrations were reached approximately at the end of the 3 h infusion and Tmax values were about 3 h across all six dose groups (**Figure 2**, **Table 4**). Once the infusion was completed, the pyragrel plasma concentration decreased rapidly, with only 1/20 Cmax concentration remaining at 4 h after initiation of the infusion. There was no statistically significant difference between pyragrel treatment groups in the dose-normalized Cmax and AUClast, indicating that a single dose of pyragrel in the range from 30 to 300 mg in healthy subjects results in a linear pharmacokinetic process. In addition, since pyragrel was rapidly metabolized to BBS and BJS after intravenous infusion, PK characteristics of the two metabolites were also considered. Both metabolites exhibited linear pharmacokinetics, and the concentration-time profiles and PK parameters of BBS and BJS showed the same trend as the parent drug across six dose groups (**Supplementary Figure 1**, **Table S2**).

**Table 4:** Pharmacokinetic parameters of pyragrel after a 3-h intravenous infusion of single-ascending-dose pyragrel (30, 60, 120, 180, 240, 300 mg) in healthy volunteers (Part I).

Parameters	Units	Single-Dose Regimen					
		30 mg (*n*=8)	60 mg (*n*=8)	120 mg (*n*=8)	180 mg (*n*=4)	240 mg (*n*=4)	300 mg (*n*=4)
t_1/2_	h	2.39 ± 1.03	4.62 ± 1.69	6.64 ± 3.53	9.64 ± 3.58	14.24 ± 1.66	9.88 ± 4.26
T_max_	h	2.75 ± 0.46	2.88 ± 0.35	2.88 ± 0.35	3.00± 0.00	2.75 ± 0.50	2.25 ± 0.50
C_max_	µg·L^-1^	368. 0 ± 91.03	711.8 ± 100.2	1,435.6 ± 319.5	2,622.5 ± 439.9	3,144.2 ± 931.2	3,011.7 ± 303.2
AUC_last_	h*µg·L^-1^	1,047.5 ± 182.7	2,003.7 ± 235.9	4,287.2 ± 812.7	7,376.5 ± 413.0	9,194.7 ± 2,703.5	9,301.4 ± 1,215.2
AUC_INF_	h*µg·L^-1^	1,051.0 ± 185.8	2,009.4 ± 235.97	4,300.5 ± 807.5	7,390.5 ± 414.7	9,224.3 ± 2,716.3	9,315.3 ± 1,219.1
Vz	L	96.16 ± 31.87	203.0 ± 81.47	278.0 ± 162.5	334.4 ± 110.8	573.6 ± 180.3	454.6 ± 191.4
Cl	L·h^-1^	29.26 ± 4.69	30.23 ± 3.58	28.76 ± 5.26	24.41 ± 1.41	27.50 ± 6.80	32.62 ± 4.29

All data are given as the mean ± standard deviation.

AUC_last_, area under the concentration-time curve from time zero to the final measurable concentration; AUC_INF_, area under the concentration-time curve from time zero to infinity; CL, clearance; C_max_, maximum concentration; t_1/2_, elimination half-life; T_max_, time to maximum concentration; Vz, apparent volume of distribution.

In terms of assessing the effect of reduced saline volume on PK profiles of pyragrel and the two metabolites in Part II, no significant difference was observed for any of the pharmacokinetic variants compared to those obtained from subjects who received pyragrel 240 mg but only diluted with 500 ml saline in Part I. The pharmacokinetic parameters and mean concentration-time profiles of pyragrel, BBS, and BJS for Part II are summarized in **Supplementary Figure 2** and **Table S3**.

**Figure 3:**
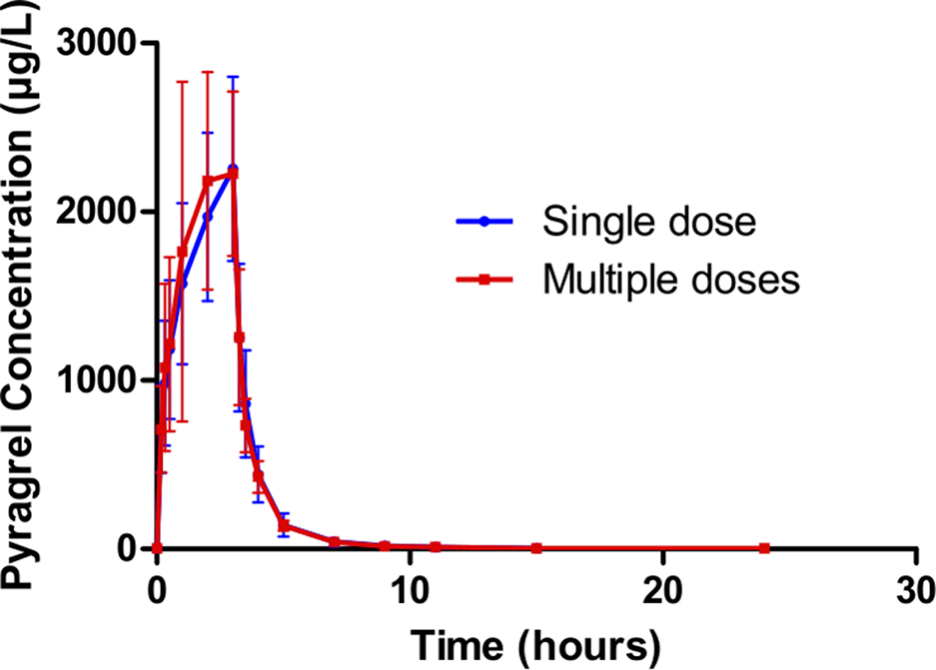
Mean (± SD) concentration-time profiles of pyragrel after i.v. 3 h infusion of multiple doses versus single dose of pyragrel 180 mg in 12 healthy volunteers (Part III). Twelve participants received multiple dose of pyragrel 180 mg for 6 days, pyragrel diluted in 250 ml saline and administrated as 3 h i.v. infusion; pyragrel concentrations of blood samples after the first dose (Day 1) and the last dose (Day 6) were determined over 24 h after the initiation of the infusion. The comparison of mean concentration-time profiles is presented in the figure.

In Part III, after repeated dose of pyragrel 180 mg for 6 days, comparison of pharmacokinetic parameters between single and multiple dose of pyragrel was presented (**Table 5**). There was a significant difference in Tmax between multiple-dose and single-dose group (p=0.025), and t_1/2_ was slightly higher than single dose but not statistically different. Except for Tmax, there were no significant differences in other key parameters between the two groups (e.g., Cmax, AUC_0-last_) (**Table 5**). The PK parameters of the two metabolites (BBS and BJS) following multiple doses of pyragrel are summarized in **Table S4** and **Figure S3**. The steady-state PK was consistent with single-dose PK characteristics, and accumulation factor of pyragrel was 1.04 after multiple doses, which demonstrated that there was no accumulation of pyragrel in plasma based on systemic exposure (**Table 5** and **Figure 3**).

**Table 5:** Pharmacokinetic parameters of pyragrel after multiple-dose versus single-dose 3-h infusion of pyragrel 180 mg in 12 healthy volunteers (Part III).

Parameters	Units	Single dose (*n*=12)	Multiple dose (*n*=12)	p
t_1/2_	h	5.12 ± 1.91	6.51 ± 2.73	0.154
T_max_	h	2.83 ± 0.39	2.42 ± 0.67	0.025*
C_min_	µg·L^-1^	4.22 ± 2.26^a^	—	—
C_avg_	µg·L^-1^	571.2 ± 171.9^b^	—	—
C_max_	µg·L^-1^	2,311.9 ± 514.4^c^	2,447.8 ± 825.3	0.593
AUC_last_	h*µg·L^-1^	6,663.3 ± 1665.7	6,909.9 ± 2066.8	0.585
AUC_INF_	h*µg·L^-1^	6,680.3 ± 1,668.0	6,939.5 ± 2,072.8	0.563
AUC_TAU_	h*µg·L^-1^	6,663.3 ± 1,665.7^e^	6,854.5 ± 2,062.7	
Vz	L	226.0 ± 141.7	28.29 ± 12.73*	0.000*
Cl	L·h^-1^	28.85 ± 8.75	28.49 ± 8.49	0.772
R^e^	1.04 ± 0.20			

^a^Steady-state trough concentration. ^b^steady-state peak concentration. ^c^steady-state average concentration. ^e^accumulation factor, R=AUC_TAU_,_multiple_/AUC_TAU_,_single_, AUC_TAU,multiple_ is area under the concentration-time curve from time zero to the end of the dosing period at steady state. AUC_TAU, single_ equal to AUC_INF_ for a single dose.

*P < 0.05 vs single administration of 180 mg of pyragrel.

**Figure 4:**
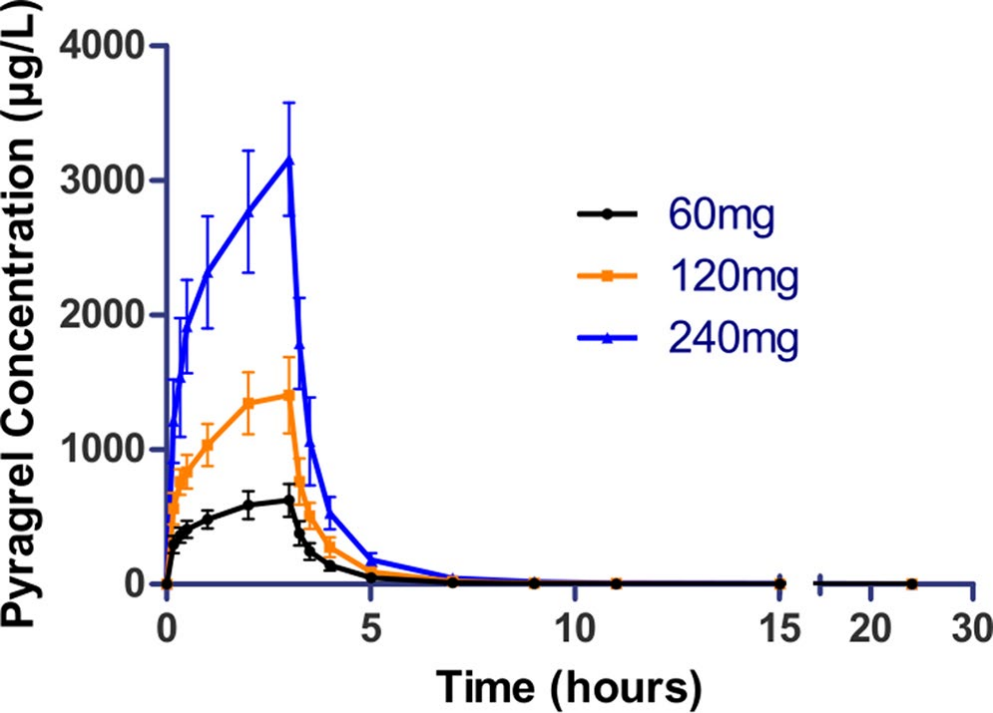
Mean (± SD) concentration-time profiles of pyragrel in a 3×3 crossover study (60, 120, or 240 mg) in 12 healthy volunteers (Part IV). Twelve participants received three doses (60, 120, or 240 mg), three period pyragrel administrations with a 7-day washout period during doses. Pyragrel diluted in 250 ml saline and infused i.v. for 3 h; pyragrel concentrations of blood samples were determined over 24 h after the initiation of the infusion at each period.

**Figure 5:**
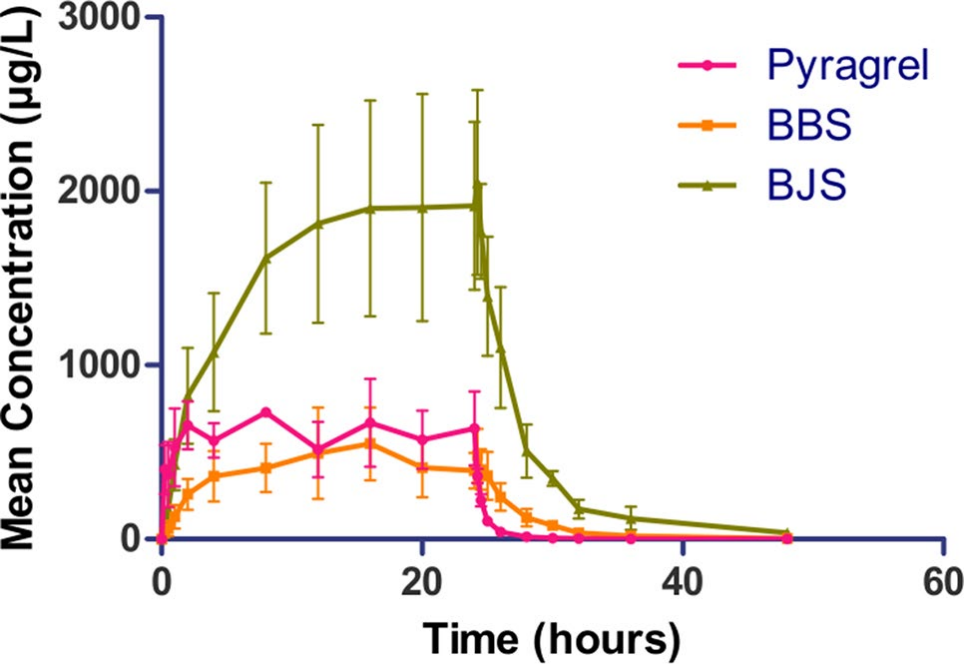
Mean (± SD) concentration-time profiles of pyragrel, BBS, and BJS after continuous intravenous infusion of 360 mg of pyragrel within 24 h in healthy volunteers (Part V). A single dose of pyragrel 360 mg was given to four participants. Pyragrel diluted in 600 ml saline and infused i.v. for 24 h; pyragrel, BBS, and BJS concentrations of blood samples were determined over 48 h after the initiation of the infusion.

In the 3×3 crossover study (Part IV), the majority of pharmacokinetic parameters showed no statistically significant difference to the corresponding dose in Part I (**Table 6**, **Figure 4**); comparable PK profiles were also observed for BBS and BJS (**Table S5**, **Figure S4**). After continuously infusing pyragrel 360 mg for 24 h (Part V), pyragrel reached the maximum concentration at 14 h and maintained the higher concentration during the remaining 10 h infusion period. The Cmax, AUClast, and AUC_INF_ were 861.67 ± 155.05 µg·L^-1^, 14,984.40 ± 2,496.64 h*µg·L^-1^, and 14,994.57 ± 2,493.9 h*µg·L^-1^, respectively. Still, the concentration decreased rapidly once the infusion was completed, with only 1/20 Cmax concentration remaining at 4 h post-infusion (**Table 7** and **Figure 5**).

**Table 6:** Summary of the pharmacokinetic parameters of pyragrel in a 3×3 crossover study (60, 120, or 240 mg) in 12 healthy volunteers (Part IV).

Parameters	Unit	Pyragrel (*n*=12)^a^		
		60 mg	120 mg	240 mg
t_1/2_	h	4.28 ± 1.84	4.82 ± 1.63	5.06 ± 1.58
T_max_	h	2.75 ± 0.45	2.58 ± 0.51	2.92 ± 0.29
C_max_	µg·L^-1^	641.2 ± 107.8	1505.6 ±178.7	3165.7 ± 426.4
AUC_last_	h*µg·L^-1^	1,990.8 ± 317.9	4,317.4 ± 541.1	9,279.1 ± 1,466.3
AUC_INF_	h*µg·L^-1^	2,003.8 ± 318.7	4,325.2 ± 541.5	9,292.8 ± 1,468.0
Vz	L	184.5 ± 80.37	197.6 ± 77.76	194.3 ± 72.28
Cl	L·h^-1^	30.65 ± 4.91	28.18 ± 3.83	26.45 ± 4.34

a Data shown as the mean ± SD.

**Table 7:** Summary of pyragrel, BBS, and BJS pharmacokinetic parameters after continuous intravenous infusion of pyragrel 360 mg within 24 h in four healthy volunteers (Part V).

Parameter	Unit	Pyragrel (*n*=4)^a^	BBS (*n*=4)^a^	BJS (*n*=4)^a^
t_1/2_	h	7.05 ± 3.74	4.74 ± 0.65	7.50 ± 2.07
T_max_	h	14.00 ± 7.66	18.06 ± 4.13	23.13 ± 2.09
C_max_	µg·L^-1^	861.7 ± 155.1	566.1 ± 195.0	2,104.0 ± 536.9
AUC_last_	h*µg·L^-1^	14,984 ± 2,497	11,500 ± 3,952	45,155 ± 12,540
AUC_INF_	h*µg·L^-1^	14,995 ± 2,494	11,533 ± 3,946	45,570 ± 12,464
Vz	L	260.2 ± 144.4	234.4 ± 89.53	92.91 ± 42.82
Cl	L·h^-1^	24.47 ± 3.75	34.11 ± 11.36	8.37 ± 2.31

a Data shown as the mean ± SD.

Additionally, urinary excretion of pyragrel and four metabolites (BBS, BJS, MW328, and MW318) were assessed in the 3×3 crossover study. The total cumulative urine excreted amounts of pyragrel as measured over 24 h in 12 subjects were 44.80, 85.07, and 175.83 mg, respectively, after single 3 h infusion of pyragrel at 60, 120, or 240 mg dose. The accumulative urinary excretion rate was 74.67%, 70.89%, and 73.26%, correspondingly. The major urinary metabolite was BJS, which accounted for ∼66%. Less than 1% of the dose was excreted as unchanged pyragrel (**Table 8**).

**Table 8:** Cumulative urinary drug recovery of pyragrel and four major metabolites (normalized to pyragrel) within 24 h after single dose of pyragrel 60, 120, or 240 mg (n=12; Mean ± SD %).

	60 mg	120 mg	240 mg
Pyragrel	0.94 ± 0.30	0.67 ± 0.14	0.79 ± 0.28
BJS	69.21 ± 10.59	66.99 ± 6.18	69.13 ± 8.39
BBS	3.90 ± 2.11	1.93 ± 0.85	2.75 ± 0.97
MW328	0.23 ± 0.33	1.16 ± 1.76	0.37 ± 0.58
MW318	0.44 ± 0.34	0.67 ± 0.25	0.44 ± 0.24
Total	74.66 ± 11.58	70.73 ± 6.76	73.28 ± 8.78

### Preliminary Pharmacodynamic Properties of Pyragrel

Given that 11-dehydrothromboxane B_2_ (11-D-HTXB_2_) is considered a reliable indicator of TXA_2_ production in humans and several animal models (Morio et al., 1993; Yamanaka et al., 1993), we preliminarily measured the production of urinary 11-D-HTXB_2_ as a maker for evaluating the TXA_2_ inhibition activity of pyragrel in three dose groups (60, 120, or 240 mg) in Part IV. 11-D-HTXB_2_ production was significantly inhibited at 4 h after pyragrel administration both in 120- and 240-mg group, and this inhibitory effect lasted for at least 12 h (P<0.05 vs preadministration). Production of 11-D-HTXB_2_ was dose-dependently inhibited by the administration of pyragrel; the urinary 11-D-HTXB2 production was 30.72 and 31.37 at 8–12 and 12–24 h period in the 240 mg group, significantly lower than that of 34.77 and 35.17 in the 60 mg group (p<0.05). (**Table 9**).

**Table 9:** Urinary 11-D-HTXB2 (pg/ml) measured in Part IV (Standardized by urine creatinine).

Group	60 mg/kg	120 mg/kg	240 mg/kg
Preadministration	38.72 ± 4.98	38.00 ± 5.18	38.37 ± 5.15
0–3 h after administration	37.83 ± 4.85	37.06 ± 5.00	36.40 ± 4.68
3–4 h after administration	36.64 ± 4.15	35.85 ± 4.40	35.37 ± 4.60
4–8 h after administration	35.05 ± 4.51	33.03 ± 5.00^▴^	33.10 ± 4.31^▴^
8–12 h after administration	34.77 ± 4.16^▴^	31.45 ± 4.88^▴▴^	30.72 ± 4.38^▴^*
12–24 h after administration	35.17 ± 4.63	32.14 ± 5.17^▴^	31.37 ± 3.79^▴▴^*

^▴^P<0.05, ^▴▴^P<0.01 vs preadministration; *P<0.05 vs 60 mg/kg.

## Discussion

The five-part trial was designed to investigate the safety, tolerability, pharmacokinetics and preliminary pharmacodynamic properties of sodium pyragrel, a new drug for the treatment of cardiovascular diseases, in 84 healthy Chinese subjects. We found that 1) Pyragrel was well tolerated at a single i.v. infusion up to 300 mg dose, multiple dose of pyragrel 180 mg and prolonged infusion of 360 mg dose within 24 h. No severe AEs occurred. AEs among different dose level of pyragrel-treated groups did not appear to be dose dependent. 2) Both pyragrel and the two metabolites (BBS and BJS) presented linear pharmacokinetics features following the dose range of 30–300 mg in the single-escalating-dosing study. Repeated dose of pyragrel 180 mg for 6 days (once- or twice-daily dosing) showed no accumulation of pyragrel, BBS, and BJS. 3) More than 70% of the total amount of drugs was excreted by urine among three groups, among which ∼66% was BJS. Less than 1% of the dose was excreted as unchanged pyragrel. 4) The production of urinary 11-D-HTXB2 was time- and dose-dependently inhibited by single infusion of pyragrel.

Sodium pyragrel (C_18_H_19_N_2_O_4_Na) is metabolized rapidly after infusion *in vivo* via double-bond reduction and double-bond oxidation, followed by glucuronide conjugation resulting in the formation of two parental structure-based metabolites, a propionic acid analog (BBS, C_18_H_22_N_2_O_4_) and a benzoic acid analog (BJS, C_16_H_18_N_2_O_4_) (Zhao et al., 2017; Yang et al., 2018). Preliminary pharmacodynamic studies showed that both BBS and BJS had weak antiplatelet and antithrombotic effects (data not shown). An acute toxicity study in mice showed relatively less toxic effect of the two metabolites; the LD_50_ values were much higher than that of 929.7 mg/kg for pyragrel. Based on the pharmacological activity, the pharmacokinetic characteristics of the metabolites were evaluated in the study. Similarly, both BBS and BJS presented linear pharmacokinetics following the administration of a single dose of pyragrel ranging from 30 to 300 mg, and no accumulation of metabolites was found with multiple-dose or prolonged continuous infusion. The urinary excretion of BJS was at least 20 times higher than BBS, possibly because of the conversion of BBS to BJS in humans, which was found previously for ozagrel metabolism (Ogiso et al., 1997).

Sodium ozagrel, the thromboxane A_2_ synthesis inhibitor used as the positive control in this study, has been demonstrated to have a significant treatment effect on acute ischemic stroke and improve the nerve dysfunction of patients with stroke when used as a monotherapy or in combination with other antiplatelet therapies (Nagatsuka et al., 1985; An et al., 2011; Zhang et al., 2012; Yang et al., 2014). Given that the recommended dose of ozagrel is 80 mg twice a day for 14 days (Wada et al., 2016) and simultaneously considering the effective dose of pyragrel in preliminary studies, we started the initial dose at 30 mg and gradually increased the dose to 300 mg to investigate the safety of pyragrel after continuous intravenous infusion for 3 h.

The data obtained in this study proved that sodium pyragrel given via single dose up to 300 mg, multiple doses of 180 mg, and even continuous infusion of 360 mg for 24 h was safe and well tolerated in healthy volunteers. No serious adverse drug reactions or severe bleeding events occurred in this study; the most common AE was an elevation in conjugated bilirubin, and the incidence is 17.6% (12 DB elevation/total 68 subjects of pyragrel). However, 25% of subjects who received ozagrel also had elevated conjugated bilirubin levels (2 DB elevation/total 8 subjects). No significant difference presented between pyragrel and ozagrel (P=0.556), indicating that DB/TB elevation was not a specific pyragrel-related side effect. Potential reasons for DB/TB elevation may involve in the pharmacological activity of pyragrel and ozagrel, or because of the unstable psychological states and changes in living conditions of participants during the trial. Other AEs included prolonged APTT, increased total bile acid levels, lower hemoglobin, palpitations, positive urinary protein, elevated alkaline phosphatase, abdominal pain, abdominal distension, positive fecal occult blood, and increased white blood cells. The frequency of these AEs was low and seemed to be dose independent.

Urinary levels of 11-D-HTXB_2_, the degree of platelet agglutination inhibition (DPAI), and the plasma levels of TXB_2_ and 6-keto-PG_1α_ are considered effective indicators to confirm the antithrombotic effect of a drug candidate (Uyama et al., 1985; Morio et al., 1993). Therefore, we determined the inhibitory effect of pyragrel on all these three markers in Part IV after a single 3 h infusion dose of pyragrel (60, 120, or 240 mg). Pyragrel slightly inhibited platelet agglutination inducted by adenosine diphosphate (ADP) and arachidonic acid (AA) (Weber et al., 1999); however, due to inter-individual variability of baseline platelet counts in whole blood and the small sample size in the trial, the results did not exhibit dose- or time-dependent pattern in our analysis (Dziewierz et al., 2015) (data not shown). Similarly, the plasma levels of TXB_2_ and 6-keto-PG_1α_ (stable metabolites of TXA_2_ and PGI_2_, respectively) were determined by ELISA since the decreased TXB_2_/6-keto- PG_1α_ ratio indicates platelet aggregation and thrombosis inhibition (Mizugaki et al., 1999). Pyragrel (60, 120, 240 mg, single i.v. dose) caused plasma 6-keto-PG_1α_ increase and plasma TXB_2_ decreased simultaneously, resulting in a moderate increase in TXB_2_/6-keto-PG_1α_ ratio, but not reaching significance (data not shown). Actually, levels of plasma TXB_2_ and 6-keto-PG_1α_ are different between patients with cerebral ischemic and healthy subjects; with the limitation of small size for pharmacodynamic activity evaluation in the study, plus the significant reduction in urinary 11-D-HTXB_2_ level here, we recommended that a larger sample size, extensive phase II study should be performed in patients to further study the pharmacodynamic characteristic of pyragrel.

## Conclusion

A single intravenous infusion of pyragrel up to 300 mg, repeated administration of 10 doses of 180 mg over 6 days, and continuous intravenous infusion of 360 mg within 24 h are safe and tolerable. Pyragrel pharmacokinetics are characterized by an approximately linear pharmacokinetic process for a single dose of 30–300 mg, rapid metabolism into BBS and BJS after a single-dose administration, and renal elimination and excretion of more than 70% of the major metabolites and some of the original pyragrel. The acceptable safety and appropriate pharmacokinetic properties of sodium pyragrel proved in this study warrant continued clinical development of pyragrel in cardiovascular patients.

## Data Availability Statement

All datasets generated for this study are included in the article/**Supplementary Material**.

## Ethics Statement

The study was conducted in accordance with the Declaration of Helsinki and the Good Clinical Practice of the International Conference on Harmonization (ICH-GCP). The study protocol and informed consent documents were approved by the Ethics Committee of the Third Xiangya Hospital of Central South University, Changsha, Hunan, China (approval number: 2013L02091 on April 17, 2014). Written informed consent was provided by all subjects prior to participating in any study-related activities in the study (Registered on China, No.: ChiCTR-IID-16010159).

## Authors Contributions

All authors contributed to drafting this manuscript by providing critical revisions and important intellectual content, and all approved the final version for submission. ZC was the medical monitor of this study. CZ and JH were involved in the design and analyzed data of the study. YH, SY, XY, and CG were responsible for the project implementation. HT, JC, and QP were involved in the analysis of blood and urine samples. GY was the principal investigator.

## Funding

This work was supported by the Program for The New Xiangya Talent Projects of the Third Xiangya Hospital of Central South University (No. 20150311) and Central South University Postgraduate Independent Exploration and Innovation Project (2017zzts221).

## Conflict of Interest

ZC was employed by Hefei Industril Pharmaceutical Institute Co., Ltd.

The remaining authors declare that the research was conducted in the absence of any commercial or financial relationships that could be construed as a potential conflict of interest.
