# Supplementary material for: Phase I Trial of Pyragrel, a Novel Thromboxane Synthetase Inhibitor, to Evaluate the Safety, Tolerability, and Pharmacokinetics in Healthy Volunteers
Source: Front Pharmacol. 2019 Oct 24;10:1231. doi: 10.3389/fphar.2019.01231 (PMC6821791; doi:10.3389/fphar.2019.01231)
Supplement: Supplementary file 1 [file DataSheet_1.docx]

Supplementary Material

Phase I Trial of Pyragrel, a Novel Thromboxane Synthetase Inhibitor, to Evaluate the Safety, Tolerability, and Pharmacokinetics in Healthy Volunteers

Chan Zou ^1#^, Jie Huang^1#^, Ye Hua^2^, Shuang Yang^1^, Xiaoyan Yang^1^, Can Guo^1^, Hongyi Tan^1^, Jun Chen^1^, Zhaoxing Chu^3^, Qi Pei^1^, Jie Huang^1*^, Guoping Yang ^1*^

^1^ Center of Clinical Pharmacology, Third Xiangya Hospital, Central South University, Changsha, China.

^2^ The First Affiliated Hospital of Xinjiang Medical University, Urumqi, Xinjiang, China.

^3^ Hefei Industril Pharmaceutical Institute Co., Ltd, Anhui, China.

# These authors contributed equally to this work

***Correspondence:**

Guoping Yang, [ygp9880@126.com](mailto:ygp9880@126.com) ; Qi Pei, [qi.pei@csu.edu.cn](mailto:qi.pei@csu.edu.cn);

**Table S1** Inclusion and exclusion criteria of study population

| Inclusion Criteria | 1. Healthy males or females between 18 and 45 years of age and no more than 15 years for the age difference for subjects within the same dose group; 2. Body weight ≥45 kg for females and ≥50 kg for males and body mass index (BMI) within the range of 19-24 kg/m^2^; 3. Subjects who were informed about the study before the experiment and signed the written informed consent voluntarily; 4. Subjects who were capable of providing written informed consent; 5. Subjects who were capable of maintaining excellent communication with investigators and completing the trial in accordance with the trial stipulations. |
| --- | --- |
| Exclusion criteria: | 1. Subjects who had undergone surgery within the last 4 weeks; 2. Subjects who were allergic to the drug or any of its excipients; 3. Subjects with a bleeding tendency, such as bleeding or subcutaneous bleeding in response to minor collisions; 4. Platelets below 20% of the normal limit; 5. History of alimentary tract hemorrhage; 6. Nervousness or anxiety; 7. History of serious diseases or circulatory, endocrine, neurological, hematological, immunological, psychiatric, metabolic, or metabolic disorders; 8. Gastrointestinal, liver and kidney diseases affecting the absorption or metabolism of drugs; 9. Laboratory examination (routine blood, routine urine, blood biochemical examination) indicating an abnormal clinical diagnosis; 10. Subjects with the following diagnoses or conditions: positive for HIV, hepatitis B surface antigen or hepatitis C virus (HCV); abnormal ECG (clinically significant), or abnormal liver function or vital signs (systolic pressure <90 mmHg or >140 mmHg, diastolic pressure <50 mmHg or >90 mmHg; heart rate <50 BPM or >100 BPM); 11. Subjects who were alcoholic or smokers; drug or alcohol dependence within the last 12 months; 12. Subjects who abused drugs; use of soft drugs (such as marijuana) with the last 3 months or hard drugs (cocaine, phencyclidine, etc.) with 1 year; 13. Food allergy or special dietary requirements; 14. Subjects who drank excessive amounts of tea, coffee and/or caffeinated beverages (8 cups or more, 250 mL/cup) daily; 15. Use of any inhibitor or inducer of hepatic function or drug metabolism (inducer examples: barbiturates, phenytoin, C Masi Bing, glucocorticoid, Omeprazole; inhibitor examples: SSRI antidepressants, cimetidine, diltiazem, macrolide, nitroimidazoles, sedatives, Vera Pammy, fluoroquinolones, antihistamines) within 30 days; 16. Use of any medication within 14 days; 17. Use of any clinical trial drug or participation in any clinical trial within 3 months prior to the trial; 18. Blood samples were taken within the last 3 months or subjects were sampled; 19. Inability to complete the study because of other reasons; 20. Other clinical reasons for unsuitable recruitment as judged by clinicians. 21. The following additional exclusion criteria (22-26) for women: 22. Use of oral contraceptives within 30 days before the test; 23. Use of long-acting estrogen or progestin injection or implant within 6 months before the test; 24. Child-bearing age combined with unprotected sexual intercourse with their partners within 14 days before the trial; 25. Specific exclusionary phases of the menstrual cycle; 26. Pregnancy and lactation. |

**Table S2** Pharmacokinetics parameters of BBS and BJS following a 3h intravenous infusion of single ascending doses of pyragrel (30, 60, 120, 180, 240, 300 mg) in healthy volunteers (Part Ⅰ).

| Parameters | Units | BBS | | | | | |
| --- | --- | --- | --- | --- | --- | --- | --- |
|  |  | 30 mg  （*n*=8） | 60 mg  （*n*=8） | 120 mg  （*n*=8） | 180 mg  （*n*=4） | 240 mg  （*n*=4） | 300 mg  （*n*=4） |
| t_1/2_ | h | 5.90 ± 2.84 | 7.94 ± 2.48 | 6.89 ± 2.29 | 7.41 ± 0.97 | 8.81 ± 0.46 | 7.85 ± 1.92 |
| T_max_ | h | 3.22 ± 0.36 | 3.25 ± 0.13 | 3.31 ± 0.18 | 3.31 ± 0.24 | 3.25 ± 0.20 | 3.19 ± 0.13 |
| C_max_ | ug·L^-1^ | 216.34 ± 48.23 | 389.37 ± 90.87 | 753.36 ± 287.7 | 1490.69 ± 358.43 | 1878.22 ± 681.05 | 2139.28 ± 589.06 |
| AUC_last_ | h*ug·L^-1^ | 1006.02 ± 209.31 | 1802.57 ± 421.18 | 3553.23 ± 1512.83 | 6542.7 ± 1174.8 | 8843.82 ± 3366.35 | 10466.78 ± 3028.35 |
| AUC_INF_ | h*ug·L^-1^ | 1016.64 ± 206.08 | 1815.58 ± 430.74 | 3566.52 ± 1518.01 | 6563.91 ± 1179.31 | 8910.06 ± 3388.83 | 10549.78 ± 3061.74 |
| Vz | L | 252.46 ± 113.75 | 377.35 ± 68.16 | 369.08 ± 168.49 | 301.81 ± 76.25 | 374.58 ± 119.99 | 345.29 ± 120.76 |
| Cl | L·h^-1^ | 30.60± 6.23 | 34.63 ± 7.86 | 37.91 ± 12.00 | 28.22 ± 5.92 | 29.59 ± 9.55 | 30.84 ± 11.23 |

| Parameters | Units | BJS | | | | | |
| --- | --- | --- | --- | --- | --- | --- | --- |
|  |  | 30 mg  （*n*=8） | 60 mg  （*n*=8） | 120 mg  （*n*=8） | 180 mg  （*n*=4） | 240 mg  （*n*=4） | 300 mg  （*n*=4） |
| t_1/2_ | h | 6.34 ± 3.26 | 9.33 ± 3.46 | 7.53 ± 0.93 | 7.13 ± 1.04 | 8.26 ± 1.25 | 7.81 ± 0.69 |
| T_max_ | h | 3.28 ± 0.34 | 3.34 ± 0.13 | 3.38 ± 0.30 | 3.38 ± 0.25 | 3.25 ± 0.20 | 3.19 ± 0.13 |
| C_max_ | ug·L^-1^ | 558.48 ± 104.12 | 1165.72 ± 341.45 | 2227.35 ± 309.61 | 3511.03 ± 81.59 | 4497.3 ± 704.77 | 5291.03 ± 1135.38 |
| AUC_last_ | h*ug·L^-1^ | 2806.28 ± 577.96 | 5666.85 ± 1847.37 | 11482.8 ± 2422.93 | 17164.86 ± 4180.39 | 21976.87 ± 3436.98 | 27384.28 ± 5489.59 |
| AUC_INF_ | h*ug·L^-1^ | 2831.08 ± 568.44 | 5720.73 ± 1872.78 | 11527.72 ± 2440.51 | 17224.23 ± 4205.43 | 22161.65 ± 3485 | 27635.25 ± 5669.44 |
| Vz | L | 99.19 ± 55.99 | 149.19 ± 60.18 | 119.39 ± 38.77 | 110.49 ± 20 | 130.04 ± 18.39 | 127.06 ± 31.44 |
| Cl | L·h^-1^ | 10.93 ± 1.91 | 11.36 ± 3.16 | 10.89 ± 2.66 | 10.93 ± 2.68 | 11.02 ± 1.64 | 11.16 ± 2.02 |

All data are given as mean ± standard deviation,

AUC_last_ area under the concentration-time curve from time zero to the final measurable concentration, AUC_INF_ area under the concentration-time curve from time zero to infinity, CL clearance, Cmax maximum concentration, t_1/2_ elimination half-life, Tmax time to maximum concentration, Vz apparent volume of distribution;

**Table S3** Summary of pyragrel, BBS and BJS pharmacokinetics parameters following a single-dose infusion of 240 mg pyragrel diluted in 250ml saline instead of 500 ml in Part I (Part Ⅱ) in healthy volunteers.

| Parameters | Units | Pyragrel （*n*=4） | BBS（*n*=4） | BJS（*n*=4） |
| --- | --- | --- | --- | --- |
| λz | ·h^-1^ | 0.13 ± 0.04 | 0.11 ± 0.03 | 0.13 ± 0.06 |
| t_1/2_ | h | 6.19 ± 2.63 | 6.31 ± 1.38 | 6.01 ± 2.31 |
| T_max_ | h | 3.00 ± 0.00 | 3.19 ± 0.24 | 3.31 ± 0.13 |
| C_max_ | ug·L^-1^ | 3360.73 ± 622.63 | 2066.22 ± 977.65 | 4488.69 ± 481.74 |
| AUC_last_ | h*ug·L^-1^ | 9600.11 ± 1446.10 | 10085.90 ± 5357.26 | 22112.64 ± 6199.19 |
| AUC_INF_ | h*ug·L^-1^ | 9638.78 ± 1487.08 | 10315.00 ± 5472.87 | 22798.13 ± 6632.76 |
| Vz | L | 221.12 ± 75.68 | 372.66 ± 424.67 | 93.92 ± 37.71 |
| Cl | L·h^-1^ | 25.38 ± 4.18 | 36.06 ± 33.86 | 11.12 ± 2.74 |
| MRT_last_ | h | 0.90 ± 0.16 | 3.50 ± 0.74 | 3.57 ± 0.85 |
| MRT_INF_ | h | 1.02 ± 0.33 | 4.13 ± 0.70 | 4.36 ± 1.08 |

**Table S4** Pharmacokinetics parameters of BBS and BJS after multiple-doses administration of pyragrel of intravenous infusion 180 mg pyragrel (Part Ⅲ) compare to single-dose of 180 mg pyragrel (Part Ⅰ) in healthy volunteers.

| Parameter | Units | BBS | | | BJS | | |
| --- | --- | --- | --- | --- | --- | --- | --- |
|  |  | Single dose | multiple dose | p | Single dose | multiple dose | p |
| t_1/2_ | h | 5.31 ± 1.42 | 6.07 ± 2.11 | 0.392 | 5.47 ± 2.33 | 5.60 ± 2.75 | 0.905 |
| T_max_ | h | 3.25 ± 0.15 | 3.19 ± 0.11 | 0.257 | 3.25 ± 0.15 | 3.23 ± 0.07 | 0.655 |
| C_max_ | ug·L^-1^ | 1324.23 ± 447.99 | 1356.91 ± 564.13 | 0.863 | 3740.95 ± 1122.76 | 3821.97 ± 1131.58 | 0.486 |
| AUC_last_ | h*ug·L^-1^ | 6022.22 ± 2291.72 | 6797.36 ± 2919.19 | 0.064 | 17281.21 ± 6010.75 | 20007.31 ± 6922.59^*^ | 0.001^*^ |
| AUC_INF_ | h*ug·L^-1^ | 6145.79 ± 2339.86 | 7027.46 ± 3017.92 | 0.034^*^ | 17737.24 ± 6144.82 | 20762.61 ± 7118.09^*^ | 0.000^*^ |
| Vz | L | 285.61 ± 221.29 | 152.37 ± 94.36^*^ | 0.045^*^ | 88.26 ± 43.45 | 48.46 ± 23.36^*^ | 0.013^*^ |
| Cl | L·h^-1^ | 36.52 ± 23.08 | 35.50 ± 18.33 | 0.765 | 11.28 ± 3.73 | 10.94 ± 3.63 | 0.277 |
| R ^d^ | 1.27 ± 0.13 | | | | 1.30 ± 0.23 | | |
| AA^e^ | 1.13 ± 0.24 | | | | 1.15 ± 0.12 | | |

*P<0.05 vs. single administration of 180 mg pyragrel

**Table S5** Summary of pyragrel pharmacokinetics parameters of BBS and BJS in a 3×3 crossover study with administration of 3 gradient doses (60, 120 or 240 mg) (Part Ⅳ) in healthy volunteers.

| Parameter | Unit | BBS（*n* = 12） | | |
| --- | --- | --- | --- | --- |
|  |  | 60 mg | 120 mg | 240 mg |
| t_1/2_ | h | 5.12 ± 1.17 | 5.08 ± 1.38 | 4.85 ± 1.28 |
| T_max_ | h | 3.29 ± 0.18 | 3.27 ± 0.17 | 3.25 ± 0.18 |
| C_max_ | ug·L^-1^ | 373.16 ± 101.22 | 837.84 ± 228.5 | 2041.56±684.12 |
| AUC_last_ | h*ug·L^-1^ | 1821.9 ± 496.75 | 3969.83 ± 1184.3 | 9440.71±3717.86 |
| AUC_INF_ | h*ug·L^-1^ | 1882.93 ± 534.37 | 4036.27 ± 1186.62 | 9572.13±3748.80 |
| Vz | L | 261.4 ± 115.98 | 250.94 ± 149.96 | 212.93 ± 126.27 |
| Cl | L·h^-1^ | 34.77 ± 11.33 | 32.54 ± 10.86 | 30.04 ± 16.36 |

| Parameter | Unit | BJS（*n* = 12） | | |
| --- | --- | --- | --- | --- |
|  |  | 60 mg | 120 mg | 240 mg |
| t_1/2_ | h | 5.57 ± 1.42 | 4.69 ± 1.32 | 4.85 ± 1.96 |
| T_max_ | h | 3.40 ± 0.23 | 3.31 ± 0.16 | 3.25 ± 0.18 |
| C_max_ | ug·L^-1^ | 1287.17 ± 355.77 | 2738.08 ± 491.68 | 5701.56±1332.92 |
| AUC_last_ | h*ug·L^-1^ | 6023.89 ± 1481.7 | 13154.85 ± 2507.72 | 27639.57±6690.24 |
| AUC_INF_ | h*ug·L^-1^ | 6224.12 ± 1468.28 | 13429.43 ± 2484.32 | 28155.99±6852.64 |
| Vz | L | 82.06 ± 29.44 | 63.45 ± 25.48 | 63.83 ± 34.04 |
| Cl | L·h^-1^ | 10.12 ± 2.29 | 9.2 0 ± 1.59 | 9.04 ± 2.42 |


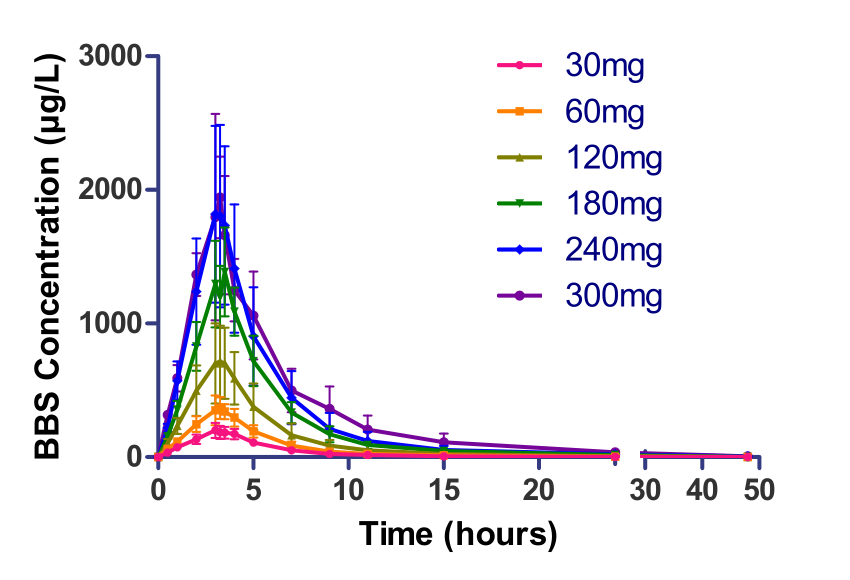

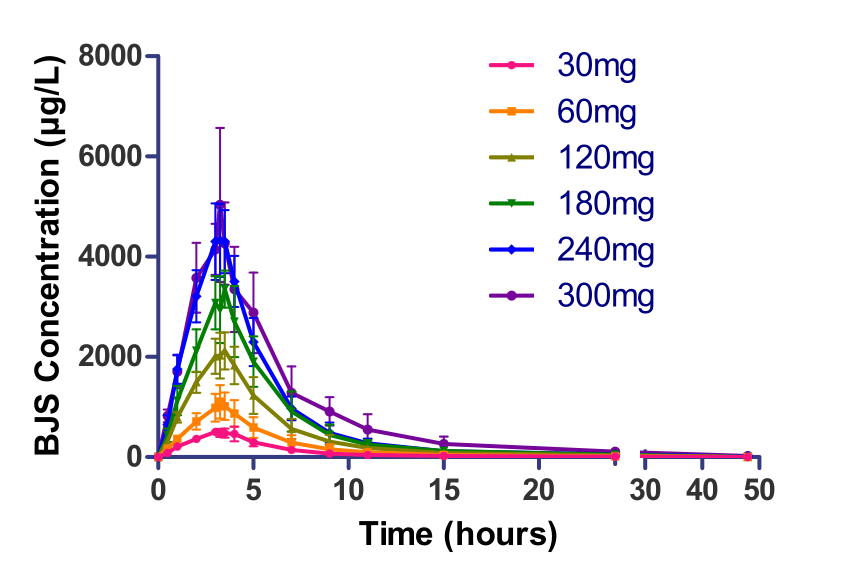


Figure S1 Mean (±SD) concentration time profiles of BBS and BJS following a 3 h intravenous infusion of single ascending doses of pyragrel (30, 60, 120, 180, 240, 300 mg) in healthy volunteers (Part Ⅰ).


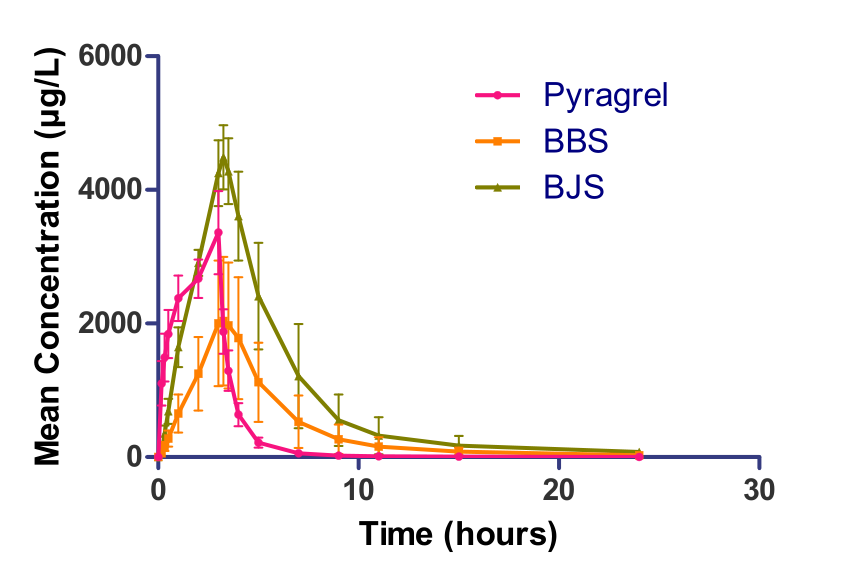


Figure S2 Mean (±SD) concentration time profiles of pyragrel, BBS and BJS following a single-dose infusion of 240 mg pyragrel diluted in 250 ml saline instead of 500 ml in Part I (Part Ⅱ) in healthy volunteers


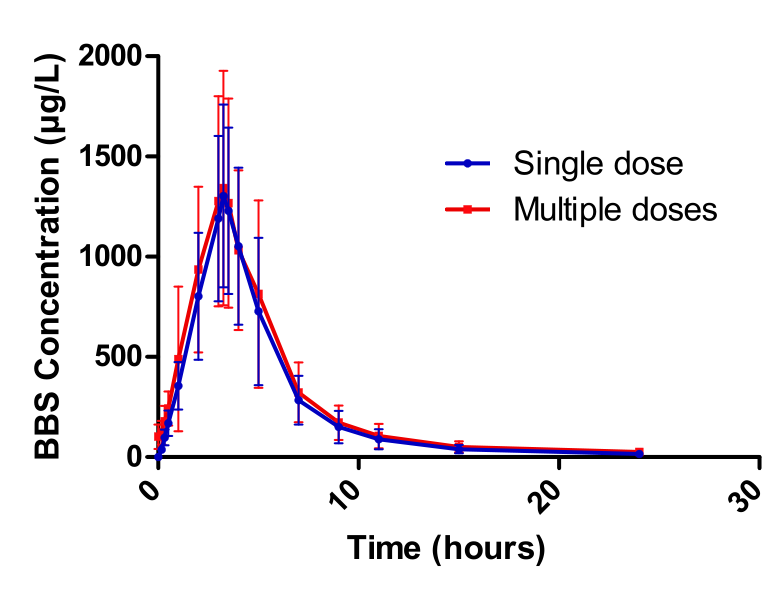

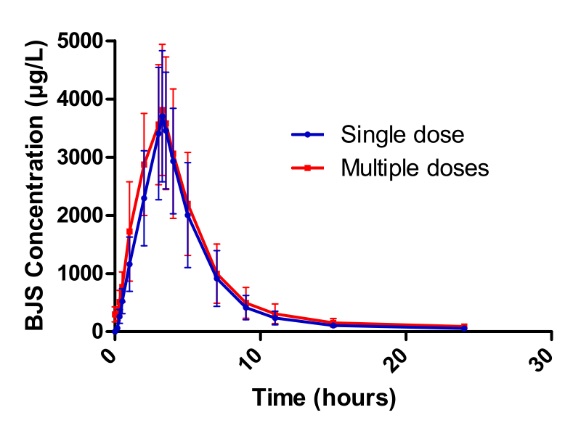


Figure S3 Mean (±SD) concentration time profiles of BBS and BJS after multiple-doses administration of intravenous infusion 180 mg pyragrel (Part Ⅲ) compare to single-dose of 180 mg pyragrel (Part Ⅰ) in healthy volunteers.


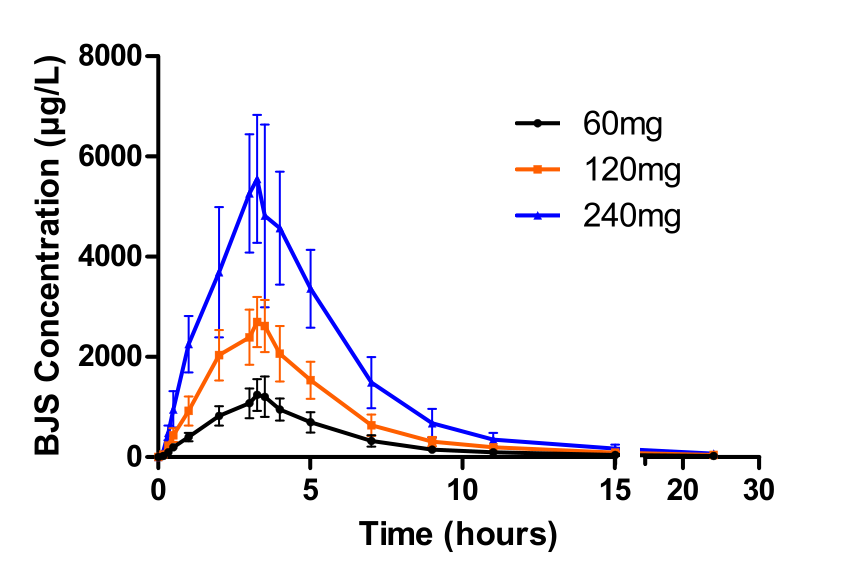

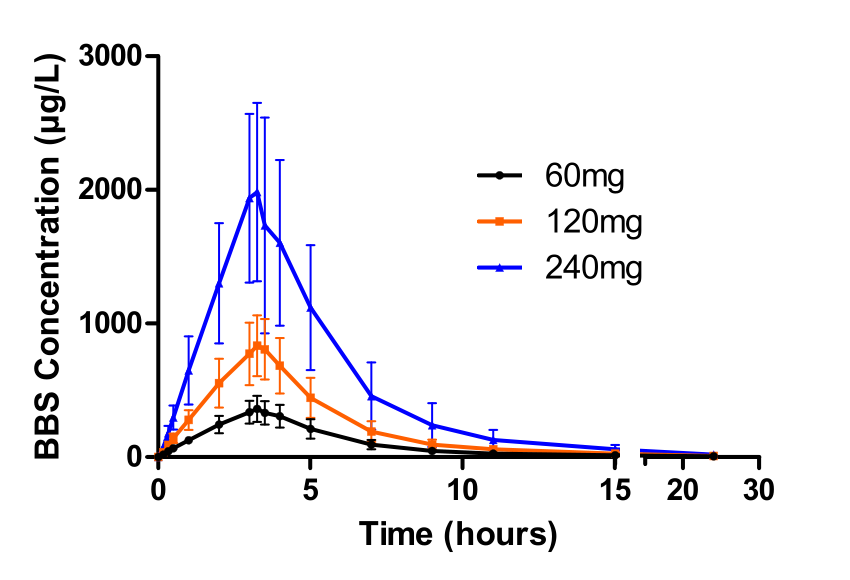


Figure S4. Mean (±SD) concentration-time profiles of BBS and BJS in a 3×3 crossover study with administration of 3 gradient doses (60, 120 or 240 mg) to further evaluate the safety and tolerability of pyragrel (Part Ⅳ) in 12 healthy volunteers.
